# COVID-19 pandemic and the reduction in ST-elevation myocardial infarction admissions

**DOI:** 10.1136/postgradmedj-2020-137895

**Published:** 2020-06-10

**Authors:** Prashanth Kulkarni, Manjappa Mahadevappa

**Affiliations:** Cardiology, CARE Hospitals, hi-tech city, Hyderabad, India; Cardiology, JSS Medical College, Mysuru, India

**Keywords:** myocardial infarction, coronary heart disease, ischaemic heart disease, hypertension, coronary intervention

Since its initial description in December 2019, the COVID-19 infection caused by severe acute respiratory syndrome coronavirus 2 has rapidly spread across the globe and according to WHO this pandemic has caused 311 847 fatalities as on 18 May 2020.^1^ With lockdown imposed in most countries of the world causing unfathomable social and economic disruption, there is palpable anxiety in the society and uncertainty in the scientific community and healthcare facilities.

The clinical presentation of COVID-19 is quite variable and still not fully elucidated. Observational studies from China revealed that 81% of the patients had mild symptoms, 14% experienced severe symptoms and 5% were critical. It has also been observed that COVID-19 infection is more likely to have poor prognosis and increased mortality in patients with advancing age, co-morbidities like obesity, chronic obstructive pulmonary disease, diabetes mellitus, hypertension, coronary artery and cerebrovascular diseases.^2  3^

Recent reports suggest that presentation of COVID-19 infection mimicking ST-elevation myocardial infarction (STEMI) with an elevation of cardiac biomarkers, electrocardiographic and echocardiographic abnormalities is highly prevalent, and is associated with more severe disease and worse prognosis.^4^ This overlapping atypical presentation of COVID-19 with STEMI has compounded the management of the infected patients and has overstretched the healthcare resources, and in some instances fatally exposed the medical personnel such as cardiologists to this contagion.

Paradoxically in cardiac centres across the world, it is observed that there has been a significant reduction in presentations of STEMI. In Spain the interventional cardiology working group of the Spanish Society of Cardiology conducted a retrospective survey at 71 cardiac service centres across the country during this on-going COVID-19 pandemic and found out that there was a significant reduction of 40% in the admissions for STEMI. The survey also revealed reductions in the number of diagnostic procedures (57%), coronary therapy (48%), structural therapy (81%) and COVID-19 infection was diagnosed in 17 interventional cardiologists (5%).^5^ In the Lombardy region, which is the epicentre of the COVID-19 outbreak in Italy, STEMI cases are down by an estimated 70%.^6^ The Minneapolis Heart Institute Foundation real-time data analysis from nine large US STEMI centres during the COVID-19 pandemic shows a 38% reduction in US cardiac catheterisation laboratory STEMI activations. This finding is consistent with the reduction reported in Spain.^7^ The reduced STEMI hospital admissions during the current COVID-19 outbreak are also being palpably felt in the Indian subcontinent.

This sudden reduction in STEMI presentations during the pandemic has intrigued cardiologists across the world. Many plausible theories are being flouted for this phenomenon. One possible explanation is that patients are too anxious to visit the hospital for fear of contracting the COVID-19 infection. Alternatively, it is also being speculated that patients with less symptoms or stable coronary artery disease (CAD) are being overlooked at the already overwhelmed hospitals that are now preferentially triaging the COVID-19 patients. These stable patients will possibly end up later in aggravated underlying CAD or delayed complications of STEMI. Other reasons proposed for a sudden reduction in STEMI admission are less exposure of the individuals to stresses like pollution, which is now substantially reduced due to lockdown imposed, no annoyance of daily commute in traffic to the workplace, less physical strain, more family time and relaxation. Also probably there is less smoking, alcohol consumption and better medication adherence leading to adequate control of hypertension and diabetes mellitus.^8^ A retrospective analysis of acute coronary syndrome (ACS) admissions in 15 Italian hospitals during the early days of COVID-19 pandemic, revealed a decrease in ACS admissions but also an increase in death rate unrelated to COVID-19 infection. This has led to doubts that few patients with ACS died without seeking medical attention.^9^ However, as STEMI is a medical emergency that forces the majority of patients to seek immediate medical help, this unusual trend is worth noticing and needs to be explored further for possible causes.

There is growing concern among cardiologists that once the COVID-19 pandemic abates there could be a sudden spurt of cardiovascular cases due to late presentation of the earlier hidden or stable patients to the hospital, and this might overwhelm the healthcare institutions leading to burnout of the medical staff.^10^ There are also apprehensions that as a consequence of this crisis, in the immediate future there could be a surge of patients with delayed mechanical complications of STEMI, reinfarctions, heart failures and arrhythmias.

The reasons for the sudden decrease in STEMI admissions need to be vigorously pursued and analysed. If this trend is genuinely due to a reduction in the daily stresses as mentioned above, it is worth the attention of policymakers, healthcare providers and cardiologists to implement preventive measures in decreasing STEMI cases and related morbidity and mortality.
